# Metastasis Suppressor microRNA-335 Targets the Formin Family of Actin Nucleators

**DOI:** 10.1371/journal.pone.0078428

**Published:** 2013-11-05

**Authors:** Jennifer Lynch, Maria H. Meehan, John Crean, John Copeland, Raymond L. Stallings, Isabella M. Bray

**Affiliations:** 1 Department of Cancer Genetics, Royal College of Surgeons in Ireland, Dublin, Ireland; 2 National Children's Research Centre, Our Lady's Children's Hospital, Dublin, Ireland; 3 School of Biomolecular and Biomedical Science, Conway Institute, University College Dublin, Dublin, Ireland; 4 Department of Cellular and Molecular Medicine, University of Ottawa, Ontario, Canada; Martin-Luther-University Halle, Germany

## Abstract

MiRNAs can have pleiotropic effects by targeting multiple genes belonging to diverse signalling networks. Alternatively, miRNAs can enhance the potency of their cellular effects by targeting multiple genes within the same genetic pathway. Previously, we and others have demonstrated that miR-335 is a potent suppressor of tumour cell migration, invasion and metastasis, in part by targeting several genes involved in these cellular processes, including *ROCK1, MAPK1, LRG1, SP1* and *SOX4*. Here, we demonstrate that direct targeting of multiple members of the formin family of actin nucleators contributes to the inhibitory effects of miR-335 in neuroblastoma cells. We demonstrate that miR-335 regulates the expression of at least five formin family members and validate three family members, *FMNL3, FMN2* and *DAAM2*, as direct targets of miR-335. The contribution of the formin family genes to cancer progression and metastasis has recently begun to emerge and here we demonstrate for the first time the ability of *FMN2* and *DAAM2* to regulate tumour cell migration and invasion, using siRNA-mediated inhibition of each of these formin genes. Finally, we demonstrate that the formin genes, in particular *FMNL3*, are responsible for the protrusion of actin-rich filopodia structures that contribute to the enhanced migratory and invasive potential associated with reduced expression of miR-335. Thus, direct targeting of the formin family contributes to the metastasis suppressing abilities of miR-335 by providing a direct regulatory link to the actin assembly machinery of the cell. We conclude that miR-335 is a master regulator of tumour cell migration and invasion by directly targeting a plethora of genes that effectively control cell migratory processes.

## Introduction

Cell migration and invasion are intrinsic to the metastatic dissemination of cancer cells and the establishment of metastatic disease, the leading cause of death among cancer patients [1], [2]. Migratory cells must undergo dynamic reconstruction of their actin cytoskeleton to provide the driving force for movement. Thus, regulation of actin remodelling activities plays a pivotal role in cancer development. The formins are a family of ubiquitously expressed and evolutionarily conserved multidomain proteins that act as potent regulators of actin dynamics through their unique ability to directly nucleate actin polymerisation, thereby regulating actin filament assembly and cytoskeleton reorganisation [3], [4], [5]. Humans have fifteen formin genes and the defining feature of each is an approximate 400 – amino acid residue FH2 domain which comprises the actin nucleation apparatus [6]. The FH2 domain associates with the free barbed end of actin filaments mediating elongation while also providing protection from capping proteins [7], [8]. This unique method of actin filament assembly facilitates the rapid formation of cellular structures comprised primarily of long unbranched actin filaments such as stress fibres and filopodia required for cell motility. Given the essential role of actin cytoskeleton rearrangements in malignant cell transformation, it is not surprising that the formin genes have been implicated in malignancy and found to be over-expressed in several cancer types including colorectal cancer, melanoma and leukaemia [9], [10], [11], [12]. Here, we demonstrate for the first time, that miR-335 regulation of cell migration and invasion in the paediatric cancer neuroblastoma can be attributed to direct targeting of formin family genes.

Neuroblastoma originates in the sympathetic nervous system and can be highly aggressive with a large proportion of children presenting with metastatic disease at the time of diagnosis [13]. The primary metastatic site is the bone marrow and the survival rate of a patient with bone metastases is merely ∼40% [14]. Understanding the molecular mechanisms and cellular processes that define metastasis is vital to improving outcome for patients with advanced stage disease. Recently, small non-coding miRNAs have emerged as key regulators of metastatic processes in addition to the plethora of gene transcripts previously identified [15], [16], [17], [18]. We have identified miR-335 as a potent suppressor of tumour cell invasion in neuroblastoma, [19] while other research groups have validated its inhibitory effect on gastric and breast cancer metastasis [20], [21]. In this study, we demonstrate a direct regulatory link between the tumour suppressor function of miR-335 and the ability of the formin genes to regulate tumour cell migration and invasion. By doing so, we expand the archive of validated miR-335 target genes to include the formin family of actin nucleators. We propose that by targeting and down-regulating the expression of at least five formin family members, miR-335 disrupts actin cytoskeleton dynamics, thereby reducing the tumour cell’s metastatic propensity.

## Materials and Methods

### Cell lines and culture conditions

SK-N-AS cells were purchased from the European Collection of Animal Cell Cultures. CHP-212 cells were purchased from the American Type Culture Collection. All cell lines were validated for the presence of previously published genomic imbalances using array comparative genomic hybridisation and by STR genotyping by the repository. Culture media was supplemented with 10% fetal bovine serum (FBS) and cells were maintained at 37 °C in a humidified incubator containing 5% CO_2_.

### Transfection procedures

Mature miR-335 mimics and scrambled oligonucleotide controls (Ambion, Austin, TX) were transiently transfected into cells at a final concentration of 30 nM by reverse tranfection using siPORT *Neo*FX reagent (Ambion). siGENOME smart pool small interfering RNAs (siRNAs) for FMNL3, FMN2, DAAM2, negative controls (Dharmacon, Chicago, IL) and co-transfection of miR-335 mimics with luciferase plasmid constructs were transiently transfected into cells using Lipofectamine 2000 (Invitrogen, Carlsbad, CA), according to the manufactor’s instructions.

### RNA isolation and quantitative real-time RT-PCR

Total RNA was isolated from cultured cells using the RNeasy Mini Kit (Qiagen, Crawley, U.K.) and quantified spectrophotometrically. Total RNA was reverse-transcribed into single-stranded cDNA using the High Capacity cDNA Reverse Transcription Kit (Applied Biosystems), as per manufacturer’s instructions. The expression of *FMNL3*, *FMN2* and *DAAM2* were quantified using the corresponding TaqMan Gene Expression Assays (Applied Biosystems) with Taqman Fast Universal PCR Master Mix (Applied Biosystems).Quantitative PCR was performed using the Applied Biosystems 7900HT Fast System. Cycle parameters for the PCR reaction were 95°C for 10 min, followed by 40 cycles at 95°C (denaturing) and annealing/extension at 60°C for 60 sec. Gene expression was normalized using endogenous 18S control. Relative fold change in expression of gene transcript was determined using the comparative cycle threshold method (2^−ΔΔct^).

### Western blotting

Total protein was extracted from cells using RIPA lysis and extraction buffer (Fisher Scientific, Hudson, NH). Protein samples were resolved by 10% SDS-PAGE and transferred to polyvinylidene difluoride (PVDF) membranes. Membranes were blocked with 5% BSA in 1X tris-buffered saline 0.1% Tween 20 (TBST) for 1 hour at room temperature. Primary antibodies for FMN1, FMN2, DAAM1, DAAM2, FHOD3 (Abcam, Cambridge, MA), FMNL3 (received as a gift from Prof. John Copeland, Ottawa, Canada) and phospho-cofilin (Cell Signalling Technology, Beverly, MA) were incubated overnight at 4°C at a dilution of 1∶1000 in 5% BSA 1X TBST. For alpha-tubulin, membranes were blocked with 5% milk 1X TBST followed by incubation with primary antibody at 1∶5000 in 5% milk 1X TBST. All membranes were incubated with either anti-rabbit or anti-mouse IgG horseradish peroxidase-conjugated secondary antibodies (Cell Signalling Technology) at 1∶5000 for 1 hour at room temperature. Blots were visualised using enhanced chemiluminescence detection system (Pierce, Rockford, IL). All Western blots were quantified by densitometric analysis performed using ImageJ software. All six formin protein sequences were aligned to each other to determine the percentage sequence similarity between all family members, this data is represented in Table S1.

### Cell migration and invasion assays

Cells transfected with siRNAs to FMNL3, FMN2, DAAM2 or negative control were transferred into 8 µm pore size transwell inserts (BD Biosciences, San Jose, CA) or Matrigel Invasion Chambers (Invitrogen) 48 hours post transfection. Cells were seeded at a density of 2.5×10^4^ for migration assays and 5×10^4^ for invasion assays. Serum-free media was used to suspend the cells in the upper chamber of the inserts and media supplemented with 10% FBS was placed in the lower chamber. Inserts were incubated at 37 °C for a 24 hour period. Following incubation, non-migratory or invasive cells were removed and migratory and invasive cells were fixed using methanol, stained with 0.5% crystal violet and counted directly by light microscopy.

### Significance testing

In order to determine if the data is normally distributed, we used the Anderson-Darling test-statistic. To carry out this analysis the following freely available test calculator was used: http://www.kevinotto.com/RSS/templates/Anderson-Darling Normality Test Calculator.xls Data was tested under the null hypothesis that each set of data is normally distributed. We determined whether to reject the null hypothesis by examining the p -value associated with each goodness-of-fit statistic. If the p-value is **less** than the predetermined critical value (we chose 0.05) the null hypothesis was rejected, and a conclusion made that the data did not come from the normal distribution. In all sample sets tested we obtained a p-value greater than 0.05 and therefore conclude that our data conforms to normal distribution. Thus, in all instances statistical significance was determined using an unpaired Student’s *t*-test. In all instances error bars represent the standard deviation between three replicate experiments unless otherwise stated.

### Actin cytoskeleton staining

Cells were seeded onto round coverslips in 6-well plates and transfected with either siRNA to FMNL3, FMN2, DAAM2 or negative control. For assessment of filopodia formation, cells were transfected with either of two FMNL3 clones, FH1-FH2 domain clone or FH1 domain only clone, as described previously [22]. Both clones were kindly gifted by Prof. Henry Higgs, New Hampshire, US. At 24 hours post-transfection cells were stained using the Actin Cytoskeleton and Focal Adhesion Staining Kit (Millipore, Billerica, MA). Briefly, coverslips containing the cells were washed with phosphate-buffered saline (PBS) followed by fixation with 4% paraformaldehyde and then permeabilised by 0.1% Triton X-100. Cells were subsequently blocked using 1% BSA for 30 minutes followed by incubation with TRITC-conjugated phalloidin for 1 hour. Finally, cells were counter stained and mounted using Vectashield mounting medium containing 4’, 6-diamidino-2-phenylindole (DAPI). Images were visualised using an LSM 700 confocal microscope (Zeiss, Jena, Germany).

## Results

### MiR-335 potentially targets six formin genes based on computational prediction

**Figure 1:**
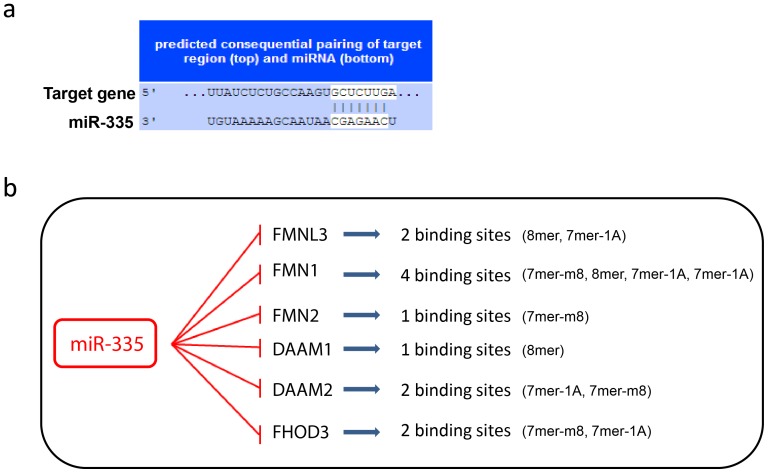
MiR-335 targeting of the formin homology family. (a) Representative eight base pair complementarity between formin target gene and miR-335. (b) The six formin genes computationally predicted to be targeted by miR-335, the number of miR-335 binding sites harboured by each gene and the seed match complementarity for each formin gene is illustrated.

According to the miRNA computational target prediction algorithm TargetScan, six formin genes contain putative miR-335 binding sites within their 3’UTR regions. Analysis of the miRNA-mRNA binding sites revealed that each of the six formin genes share the same eight base pair seed sequence complementarity with miR-335 (Figure 1a). Moreover, as illustrated in Figure 1b many of the genes possess multiple binding sites for miR-335 for example; *FMN1* harbours four miR-335 binding sites. Multiple miRNA binding sites may facilitate more stringent regulation of target gene expression. The fact that miR-335 targets almost half the members of the formin homology family suggests that their regulation may be an important function of miR-335 activity and contributes to its role as a metastasis suppressor miRNA.

### Formin expression levels are regulated by miR-335 in neuroblastoma cells

**Figure 2:**
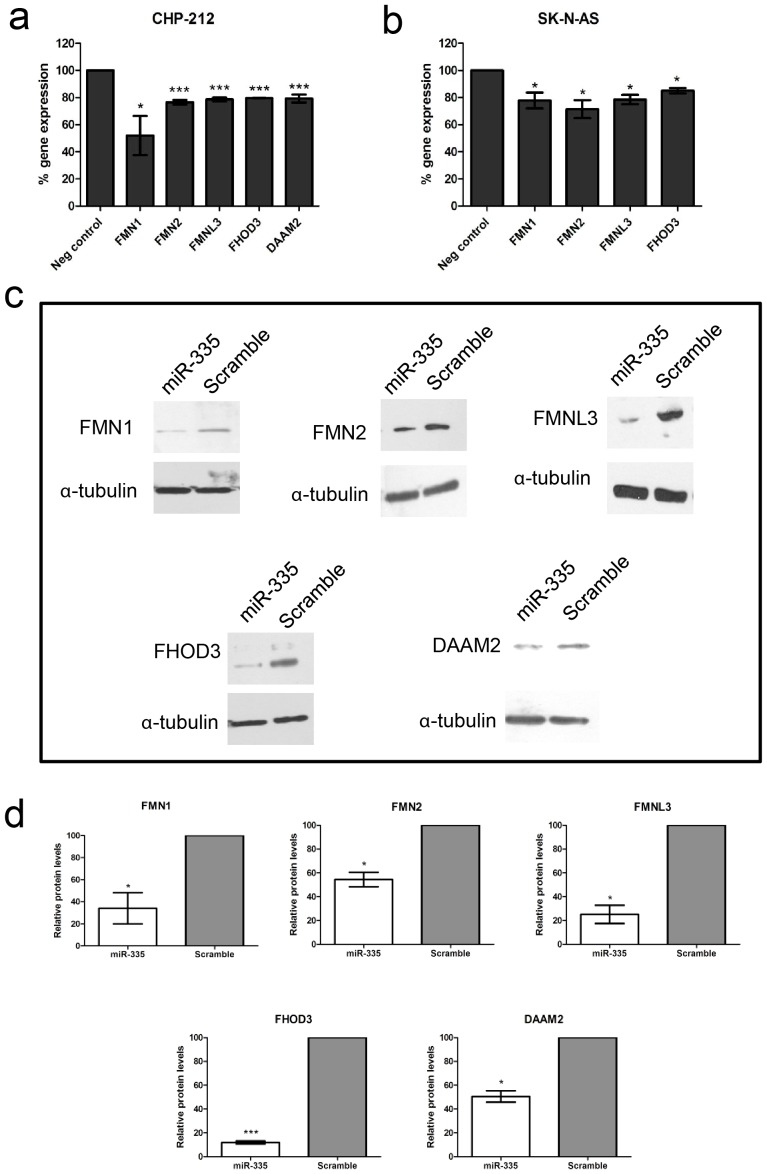
Regulation of formin gene expression by miR-335 in neuroblastoma cells. Reverse transcription-qPCR expression analysis of the potential formin target genes following transfection with miR-335 mimics in (a) CHP-212 and (b) SK-N-AS cells. Low endogenous expression of *DAAM2* resulted in unreliable quantification in SK-N-AS cells. (c) Reduced expression of all five formin genes was revealed at protein level by Western blot analysis. (d) Percentage reduction in protein expression for each of the five formin genes is illustrated as quantified by densitometric analysis of duplicate blots for each gene, normalised against the corresponding alpha-tubulin controls. Asterisks indicate statistical significance obtained using an unpaired Student’s *t*-test. *P<0.5, **P<0.005, ***P>0.0005.

To determine if the six formin genes were biologically relevant targets of miR-335 in neuroblastoma cells, two cell lines CHP-212 (MYCN amplified) and SK-N-AS (MYCN non-amplified) were transfected with mature miR-335 mimics and the resultant alterations in formin gene expression were analysed by real-time qPCR 48 hours post-transfection. The expression of five formin genes (*FMNL3, FMN2, FMN1, FHOD3, DAAM2*) were significantly reduced following miR-335 up-regulation in CHP-212 cells (Figure 2a). Significant reductions in RNA expression were observed for four formin genes following transfection with miR-335 mimics in SK-N-AS cells (*FMNL3, FMN2, FMN1, FHOD3)* (Figure 2b). Low endogenous expression of *DAAM2* in SK-N-AS prevented accurate quantification by RT qPCR in this cell line. DAAM1 expression was undetectable in both neuroblastoma cell lines indicating that it is potentially not expressed in these cells. Analysis of formin expression at protein level revealed diminished protein expression for all five formin genes as assessed by Western blot 72 hours post-transfection in CHP-212 cells (Figure 2c). Statistically significant reductions in protein expression were verified by densitometric analysis performed on duplicate experiments (Figure 2d). Representative Western blots are displayed in full in Figure S1.

### *FMNL3*, *FMN2* and *DAAM2* are direct targets of miR-335

**Figure 3:**
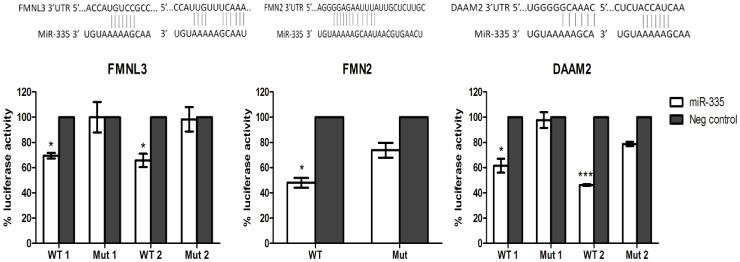
*FMNL3, FMN2* and *DAAM2* are direct targets of miR-335. Luciferase reporter assays were performed to confirm direct targeting of *FMNL3, FMN2* and *DAAM2* by miR-335. The miRNA:mRNA sequence complementarity is also illustrated for each of the three genes. *P<0.5, **P<0.005, ***P>0.0005.

To verify that miR-335 regulation of formin gene expression is mediated by a direct rather than indirect mechanism, luciferase reporter assays were performed. Three genes (*FMNL3, FMN2* and *DAAM2*) were chosen for direct targeting verification. These genes were selected to represent three different subfamilies of the formin homology family. Luciferase reporter plasmids were constructed for *FMNL3, FMN2* and *DAAM2* containing the miR-335 binding sites located within the 3’UTR of each gene (Figure S2). *FMN2* possesses a single binding site for miR-335 whereas *FMNL3* and *DAAM2* each contain two miR-335 binding sites. The miR-335 binding sites are located at a distance greater than 500 bp apart and therefore separate wild-type and mutant reporter constructs were created for each individual binding site in the case of *FMNL3* and *DAAM2*. Co-transfection of the reporter construct with miR-335 mimics resulted in significantly reduced luciferase activity for the wild-type compared to mutant constructs for all three genes (Figure 3). This provides confirmation that *FMNL3, FMN2* and *DAAM2* are all directly regulated by miR-335.

### siRNA knockdown of *FMNL3*, *FMN2* and *DAAM2* significantly reduces the motile and invasive capacity of neuroblastoma cells

**Figure 4:**
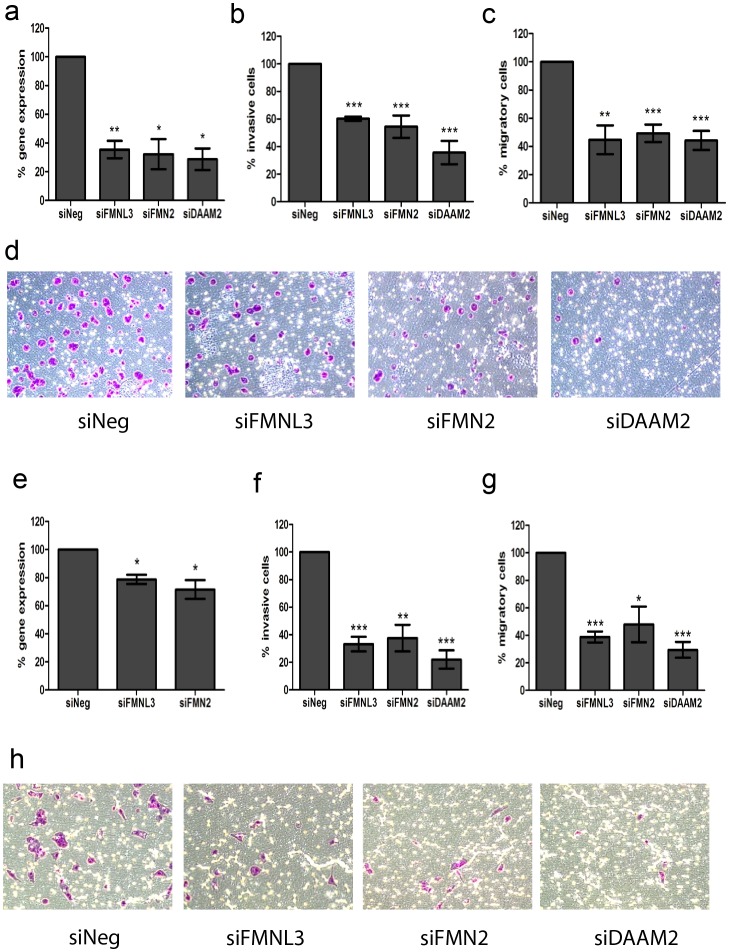
Reduced cell migration and invasion is a consequence of siRNA-mediated inhibition of *FMNL3, FMN2* and *DAAM2* expression. (a) Reverse transcription-qPCR was performed to confirm significant reductions in the mRNA levels of the three formin genes in CHP-212 cells. Subsequently, siRNA-mediated knockdown of each of the three formin genes resulted in significant reductions in CHP-212 cell migratory (b) and invasive (c) potential as measured by their ability to migrate across a non-Matrigel membrane and a Matrigel-coated membrane, respectively. (d) Representative images of the reduced invasive potential of CHP-212 cells following siRNA knockdown of *FMNL3, FMN2* and *DAAM2* as assessed by their abitility to migrate across a Matrigel-coated membrane. (e) Significant reductions in mRNA expression were also verified in SK-N-AS cells following siRNA knockdown. Low endogenous expression of *DAAM2* in SK-N-AS cells prevented accurate quantification of knockdown in this cell line. Subsequent reductions in migration (f) and invasion (g) were also observed in SK-N-AS cells. (h) Representative images of reduced cell invasion in SK-N-AS cells following siRNA knockdown of the three formin genes. *P<0.5, **P<0.005, ***P>0.0005.

We previously demonstrated that miR-335 is a potent suppressor of neuroblastoma cell migration and invasion [19]. Therefore, we reason that if miR-335 regulation of formin gene expression is of significant biological relevance in neuroblastoma cells, independent inhibition of formin gene expression should influence neuroblastoma cell migration and invasion capacity. siRNA-mediated knockdown of *FMNL3, FMN2* and *DAAM2* was confirmed by real-time qPCR in CHP-212 cells 24 hours post-transfection. RNA expression for each of the three genes was reduced by approximately 60% (Figure 4a). Significant reductions in protein expression for all three genes were also verified by Western blot analysis (Figure S3) (full images of representative blots displayed in Figure S4). Subsequently, CHP-212 cells transfected with the siRNA for each of the three formin genes or a siRNA negative control were trypinised, counted and seeded into Matrigel-coated invasion chambers for a 24 hour period. As illustrated in Figure 4b, inhibition of *FMNL3, FMN2* and *DAAM2* significantly reduced the motile capacity of CHP-212. Similarly, siRNA knockdown of the formins significantly diminished *in vitro* invasive potential (Figure 4c and d). siRNA-mediated inhibition studies were also conducted in SK-N-AS cells wherein significant suppression of gene expression was confirmed by real-time qPCR (Figure 4e). As discussed previously, SK-N-AS cells possess low endogenous expression of *DAAM2* which prevented accurate verification of siRNA-mediated knockdown by real-time qPCR in this cell line. Diminished cell migration (Figure 4f) and invasion (Figure 4g and h) were demonstrated by cells transfected with siRNAs to *FMNL3, FMN2* and *DAAM2* (Figure 4f).

### MiR-335 up-regulation and siRNA knockdown of *FMNL3*, *FMN2* and *DAAM2* disrupt actin cytoskeleton dynamics

**Figure 5:**
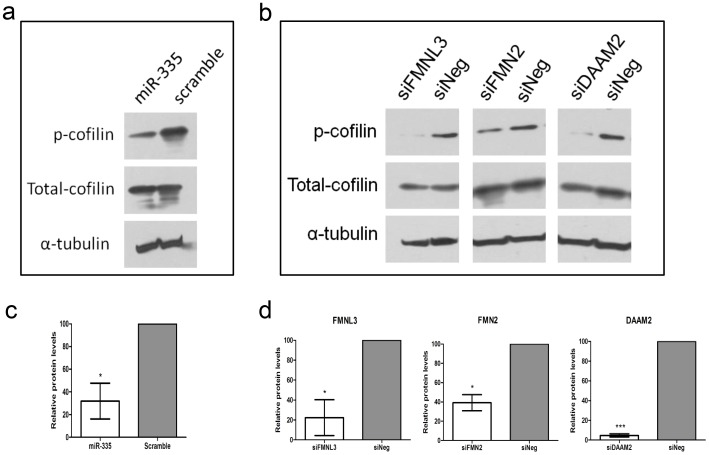
MiR-335, FMNL3, FMN2 and DAAM2 regulate actin cytoskeleton dynamics. (a) Transfection of cells with miR-335 mimics resulted in a reduction in the levels of phosphorylated cofilin protein and did not affect the levels of total cofilin protein (b) siRNA-mediated inhibition of *FMNL3, FMN2* and *DAAM2* produced a reduction in phosphorylated cofilin levels without altering total cofilin protein levels. Densitometric quantification of reduced phospho-cofilin protein in response to enhanced miR-335 expression (c) and reduced expression of *FMNL3, FMN2* and *DAAM2* (d) performed on duplicate blots and normalised against the appropriate alpha-tubulin loading control.

Cofilin, a member of the actin depolymerisation factor (ADF) family, plays a key role in controlling actin filament dynamics and reorganisation by depolymerising and severing actin filaments [23], [24]. Given its role as a critical regulator of actin dynamics, cofilin can be used as a marker for assessing alterations to actin cytoskeleton dynamics. The actin depolymerising activities of cofilin are subject to negative regulation by phosphorylation of Ser-3 by LIM kinase [25], [26]. The phosphorylation status of cofilin was assessed to determine if actin cytoskeleton dynamics were disrupted as a consequence of altered miR-335 expression and subsequent formin target gene expression. CHP-212 cells were transfected with miR-335 mimics and the phosphorylation levels of cofilin were analysed by Western blot 24 hours post-transfection using a phospho antibody specific to residue Ser-3 of cofilin. As illustrated in Figure 5a, miR-335 up-regulation stimulated a resultant reduction in the levels of phosphorylated cofilin protein compared to negative control cells, while not affecting total levels of cofilin protein. Correspondingly, siRNA-mediated knockdown of each of the three formin genes *FMNL3, FMN2* and *DAAM2* also resulted in reduced phosphorylation levels of cofilin (without affecting total protein levels), compared to negative control cells 24 hours post-transfection (Figure 5b). Significant reductions in phosphophorylated cofilin levels were quantified by densitometric analysis as displayed for miR-335 transfected cells (Figure 5c) and cells transfected with siRNAs to *FMNL3, FMN2* and *DAAM2* (Figure 5d), full western blot images displayed in Supplemental Figure S5. This would suggest that disruption to actin dynamics contributes to the reduced migratory capacity associated with the up-regulated expression of miR-335 and down-regulated expression of *FMNL3, FMN2* and *DAAM2*.

### Over-expression of *FMNL3* potently induces filopodia formation

**Figure 6:**
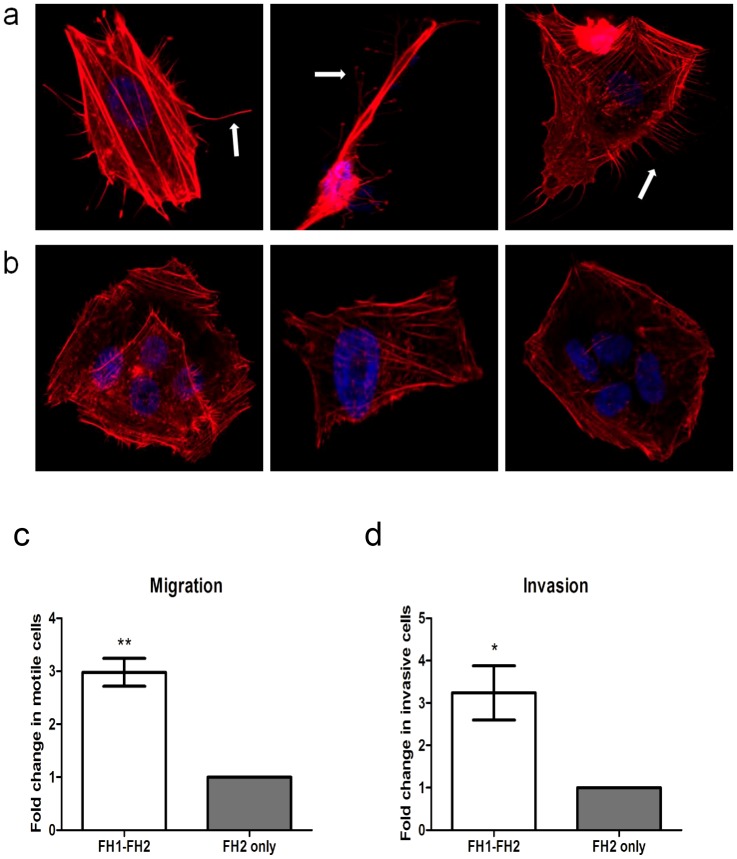
FMNL3 nucleates filopodia formation. (a) SK-N-AS cells transfected with FH1-FH2 domain FMNL3 clone display abundant filopodia protruding from the surface of the cells. (b) Cells transfected with the FH2 domain only FMNL3 clone do not display filopodia protrusions. TRITC-conjugated phalloidin (red) stains F-actin and DAPI (blue) stains nuclei. (c) SK-N-AS cells transfected with the FH1-FH2 FMNL3 clone displayed significantly enhanced migratory potential compared to cells transfected with the FH2 domain in isolation. Cells displayed no significant alteration in migratory potential when transfected with either the FH1-FH2 or FH2 domain clones in conjunction with either miR-335 or scramble control. (d) Cells transfected with the FH1-FH2 FMNL3 clone also demonstrated increased invasive potential compared to the FH2 domain in isolation. Cells displayed no significant alteration in invasive capacity when transfected with either the FH1-FH2 or FH2 domain clones in conjunction with either miR-335 or scramble control.

The protrusion of thin, actin-rich finger-like structures termed filopodia, from the surface of a cell, is one of the defining features in the acquirement of invasive capabilities. Our previous investigation of the effect of miR-335 on actin cytoskeleton dynamics revealed the protrusion of abundant filopodia from the surface of the cell in response to reduced expression of miR-335 [19], consistent with the observed enhancement in migratory and invasive potential of these cells. The formin family of proteins represent attractive candidate target genes through which reduced expression of miR-335 results in filopodia formation. In particular, FMNL3 has been demonstrated to nucleate filopodia formation [22] and was, therefore, selected to investigate filopodia formation in neuroblastoma cells. Harris *et al.*, have previously demonstrated that the FH2 domain of FMNL3 is capable of nucleating filopodia formation only in conjunction with the FH1 domain. The FH1-FH2 domain and FH2 domain only FMNL3 clones were kindly gifted by Prof. Henry Higgs (New Hampshire, US) transfected into SK-N-AS neuroblastoma cells and filopodia formation was assessed by immunofluorescent staining of the actin cytoskeleton. As illustrated in Figure 6, over-expression of the FH1-FH2 domains of FMNL3 potently induced the protrusion of filopodia from the cell surface. Consistent with findings by Harris *et al.*, over-expression of the FH2 domain in isolation failed to produce filopodia formation [22]. Furthermore, the migratory and invasive capacity of SK-N-AS neuroblastoma cells were assessed following transfection of either the FH1-FH2 or FH2 FMNL3 domain clones. Indeed, the cells displayed significant enhancement of their migratory (Figure 6c) and invasive (Figure 6d) potential when over-expressing the FH1-FH2 domains of FMNL3 in comparison to those over-expressing the FH2 domain of FMNL3 in isolation. To serve as an additional control, cells were co-transfected with the FH1-FH2 clone in addition to either miR-335 or scramble control. As expected, co-transfection with miR-335 had no significant effect on cell migration or invasion when compared to co-transfection with scramble control. This is due to the fact that miR-335 cannot target and down-regulate the FH1-FH2 domain of FMNL3 and down-regulating endogenous FMNL3 levels is overcome by the extensive over-expression by the FH1-FH2 clone (Figure S6). Therefore, low expression of miR-335 leads to the enhanced expression of the formin genes, in particular FMNL3, which nucleates the formation of abundant filopodia protrusions thereby enhancing the cell’s migratory and invasive potential.

## Discussion

MiRNAs have recently received recognition for the important role that they play in the complex circuitry that regulates cancer metastasis. Of these, miR-335 has been identified as a potent suppressor of tumour cell migration and invasion in multiple forms of cancer, including our previous work which demonstrated that miR-335 can indirectly regulate the myosin-driven motor activity of the cytoskeleton by targeting the *ROCK1, MAPK1* and *LRG1* gene transcripts. [19]. In the present study, we demonstrate that miR-335 can in fact also directly regulate actin filament assembly and disassembly by targeting the formin family of actin nucleators. The formin family members all function to nucleate linear unbranched actin for the formation actin filaments that comprise distinct cellular structures such as stress fibres, filopodia, actin cables and actin-rich adhesion junctions [27], [28]. Furthermore, the formins are the only nucleators that are themselves fully competent to nucleate actin filament assembly. Given the potency of formin mediated actin nucleation and assembly, their activity must be tightly regulated within the cell. Indeed, the fact that mammalian cells encode such numerous formin genes (fifteen in total) is believed to relate to the requirement for multiple means of regulation for this important family of actin nucleators. In this study, we illustrate that miR-335 targets at least five family members as a means of establishing effective control over formin-mediated actin nucleation.

To our knowledge there is currently only one study in the literature regarding the regulation of the formins by miRNAs in which the researchers validate that FMNL2 is regulated by miR-137 in colorectal cancer cells [29]. We demonstrate, by Western blot analysis, that miR-335 regulates the expression of at least five formin family members, three of which we validate as direct targets of miR-335: *FMNL3, FMN2* and *DAAM2*. Furthermore, we confirm that inhibiting the expression of *FMNL3, FMN2* and *DAAM2* significantly reduces the motile and invasive capacity of neuroblastoma cells. This would suggest that down-regulating the expression of the formin homology family contributes to the potency of miR-335 mediated suppression of migration and invasion.

MiR-335 suppression of cell migration mediated through formin inhibition is presumably accomplished by disrupting actin cytoskeleton dynamics. In an effort to assess any disruption to actin dynamics we analysed the activity of cofilin, a marker of actin cytoskeleton dynamics. Owing to its actin depolymerising and severing activities cofilin is a key regulator of actin cytoskeleton dynamics and has been identified as a determinant of metastasis [30]. The actin depolymerising activity of cofilin is inhibited by LIM kinase phosphorylation on Ser-3 [25], [26]. However, it would appear that the regulation of cofilin activity is a complex cell type and context dependent process. In some cell types cofilin is mostly phosphorylated in resting cells, with subsequent induction of motility resulting in dephosphorylation and activation of cofilin [31], [32]. In contrast, it has been found that cell stimulation with epidermal growth factor (EGF) leads to rapid phosphorylation of cofilin [33]. Here, we demonstrate that a reduction in cell migratory and invasive ability mediated by both miR-335 over-expression and siRNA-mediated knockdown of *FMNL3, FMN2* and *DAAM2* is accompanied by a reduction in the phosphorylated levels of cofilin. Our results are in agreement with similar findings in neuroblastoma cells by other researchers. For example, Meyer *et al*, established that the enhanced motility of neuroblastoma cells induced by stimulation with insulin-like growth factor (IGF) is accompanied by an increase in phosphorylated cofilin [34]. Takemura *et al*, demonstrated that calcium induced neurite outgrowth, a process that requires extensive actin remodelling, leads to enhanced cofilin phosphorylation [35].

A critical hallmark in the remodelling of the actin cytoskeleton to facilitate acquisition of metastatic potential is the expression of abundant exploratory, sensory organelles, termed filopodia [36]. These are thin, needle-like protrusions from the cell’s surface that are composed of parallel bundles of filamentous actin. Our previous investigation of the effect of miR-335 on actin cytoskeleton dynamics revealed the protrusion of abundant filopodia from the surface of the cell in response to reduced expression of miR-335 [19], consistent with the observed enhancement in migratory and invasive potential of these cells. The extension of filopodia pushes the cell’s leading edge forward and thereby promotes directed migration [37]. The actin nucleating ability of the formins contributes to filopodia formation. In particular, mDia2, FMNL3 and FMNL2 have been documented to induce the formation of these protruding structures in mammalian cells [22], [38], [39]. In this study, we validate the ability of FMNL3 to induce filopodia in neuroblastoma cells and furthermore, implicate FMNL3-induced filopodia formation in the enhanced migratory and invasive potential of cells with reduced miR-335 expression.

Our previous work identified that low expression of miR-335 correlates with unfavourable metastatic disease and poor patient survival in neuroblastoma [19]. Here, we postulate that low expression of miR-335 leads to the enhanced expression of five formin family members whose activity as potent regulators of actin dynamics may contribute to the poor survival phenotype of these tumours by enhancing the metastatic processes of migration and invasion. Although not tested directly in this study, in a true biological environment it is believed that different members of the formin family play distinct roles in regulating discrete aspects of the cell migratory process. Indeed, cell migration and invasion is a complex process requiring coordinated cross talk between distinct cellular components such as the microtubule network, the actin cytoskeleton network and the large network of adhesion junctions. The precise role of many of the formin genes in regulating the overall process of cell migration is yet to be deciphered. Here, we have demonstrated that FMNL3 regulates filopodia formation which contributes to the migratory potential of the cells. FMN1, on the other hand, has been shown to be recruited to adhesion junctions by α-catenin in epithelial cells and assembles radial actin cables required for cell adhesion [40]. In a true biological environment and during the complex process of tumourigenesis and cancer progression it is likely that miR-335 regulation of multiple formin family members is a highly coordinated process in which it harnesses control of filopodia formation, actin stress fibre contraction and formation and release of adhesion junctions to ultimately accomplish coordinated control of cancer cell migration and invasion. Due to the pleiotropic nature of miRNA activity, a single miRNA is capable of simultaneously regulating the expression of multiple target genes and signalling networks, thereby controlling multiple aspects of malignant cell transformation. Here, we exemplify the ability of miRNAs to concentrate their effects in this manner by demonstrating that miR-335 targets multiple members of the metastasis regulating formin family.

## Supporting Information

Figure S1 **Full Western blots for data shown in** **Figure 2** **.** Molecular weight standards are displayed to the left of each blot. Each blot was subsequently striped and re-probed with alpha-tubulin (∼50 kDa) to serve as a loading control for normalisation and quantification of results. (S1a) CHP-212 cells transfected with either miR-335 or scramble control and probed with anti-FMN1 antibody which detects a band of approximately 85 kDa. (S1b) Full blot for FMN2 protein (∼67 kDa) and corresponding alpha-tubulin. (S1c) MiR-335 and scramble control transfected protein displaying reduction in FMNL3 protein levels (∼37 kDa). (S1d) Full blot image of FHOD3 protein (∼150 kDa) and corresponding alpha-tubulin control. (S1e) Western blot of DAAM2 protein (∼100 kDa) and equivalent loading control.
(TIF)

Figure S2 **3’UTR sequence inserts for** ***FMNL3, FMN2*** **and** ***DAAM2*** **.** The miR-335 seed sequence match or mutated seed match are highlighted in red.
(TIF)

Figure S3 **Western blot verification of siRNA knockdown of FMNL3, FMN2 and DAAM2.** siRNA-mediated knockdown of FMNL3, FMN2 and DAAM2 produced significant reductions in the protein levels of each of the three formin genes by 72 hours post-transfection. Western blots were quantified by densitometric analysis of duplicate experiments.
(TIF)

Figure S4 **Full Western blot images corresponding to data displayed in Figure S3.**
(TIF)

Figure S5 **Full Western blot images for data displayed in** **Figure 5** **. (**S5a) Full blots of cells transfected with miR-335 or scramble control and analysed for levels of phosphorylated cofilin (∼19 kDa), subsequently re-probed for total cofilin protein levels (∼19 kDa) and finally re-probed for alpha-tubulin levels (∼50 kDa). (S5b) Cells transfected with siRNA to *FMNL3* or siNegative control and analysed for phosphorylated cofilin, total cofilin and alpha-tubulin protein levels. (S5c) Cells transfected with siRNA to *FMN2, DAAM2* or siNegative control and analysed for phosphorylated cofilin, total coffin and alpha-tubulin protein levels.
(TIF)

Figure S6 **Analysis of co-transfection of FH1-FH2 FMNL3 in conjunction with miR-335 on cell migration and invasion.** Co-transfection of SK-N-AS cells with the FH1-FH2 clone in addition to either miR-335 or scramble control had no significant effect on cell migration (a) or cell invasion (b).
(TIF)

Table S1 Analysis of the specificity of the formin antibodies. (a) The accession number and immunogen sequence for each of the antibodies is displayed, the immunogen sequence for FMNL3 antibody was unattainable. The immunogen sequence of each of the formin antibodies was aligned to each of the five other formin member proteins. In the case of FMNL3, the alignment of the entire FMNL3 protein sequence with all five other protein members was analysed. None of the sequence alignments produced either continuous stretches of 9–13 or discontinues stretches of 15–22 amino acid complementarity, the defining criteria for peptide immunogenicity [41]. The only alignments that produced the required continuous amino acid complementarity for successful immunogenicity were DAAM1-DAAM2 sequence alignments. (b) The protein sequences of all six formin family members were aligned to each other and the percentage sequence similarity between each individual pair was calculated.
(TIF)
