# Correction: Metastasis Suppressor microRNA-335 Targets the Formin Family of Actin Nucleators

**DOI:** 10.1371/journal.pone.0295370

**Published:** 2023-12-14

**Authors:** Jennifer Lynch, Maria H. Meehan, John Crean, John Copeland, Raymond L. Stallings, Isabella M. Bray

This Correction addresses errors in Figs 2 and 5.

**Fig 2:**
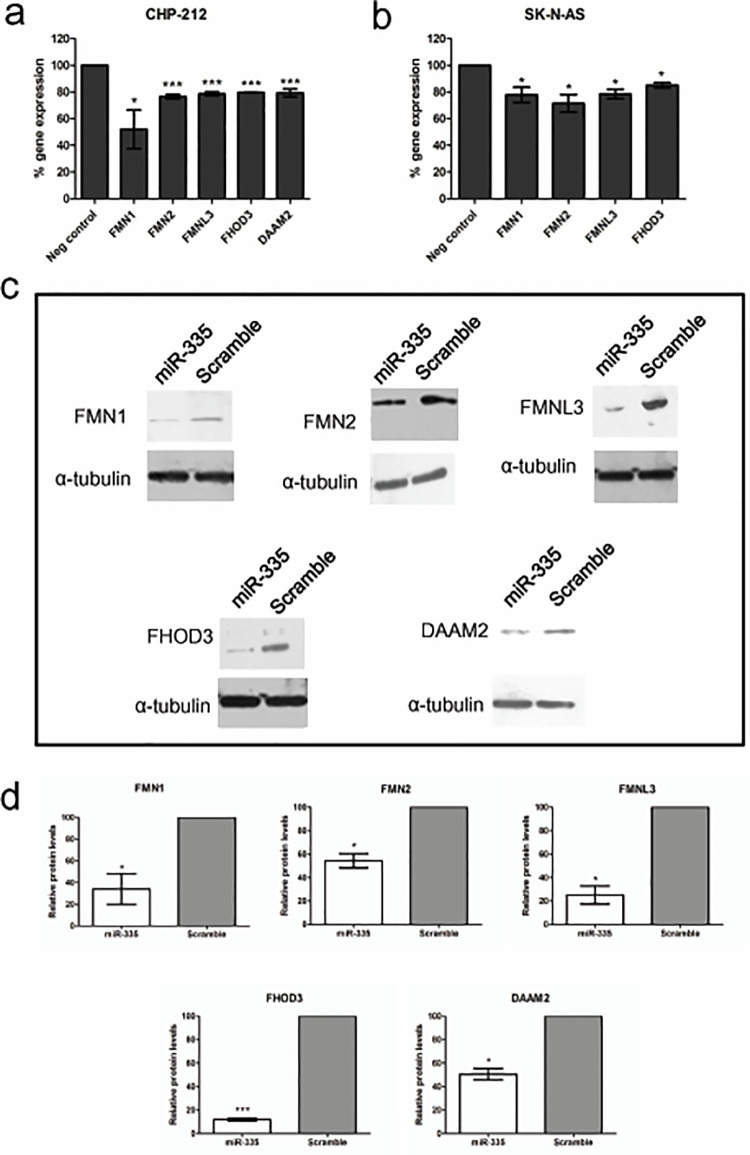
Regulation of formin gene expression by miR-335 in neuroblastoma cells. Reverse transcription-qPCR expression analysis of the potential formin target genes following transfection with miR-335 mimics in (a) CHP-212 and (b) SK-N-AS cells. Low endogenous expression of *DAAM2* resulted in unreliable quantification in SK-N-AS cells. (c) Reduced expression of all five formin genes was revealed at protein level by Western blot analysis. (d) Percentage reduction in protein expression for each of the five formin genes is illustrated as quantified by densitometric analysis of duplicate blots for each gene, normalised against the corresponding alpha-tubulin controls. Asterisks indicate statistical significance obtained using an unpaired Student’s *t*-test. *P<0.5, **P<0.005, ***P>0.0005.

**Fig 5:**
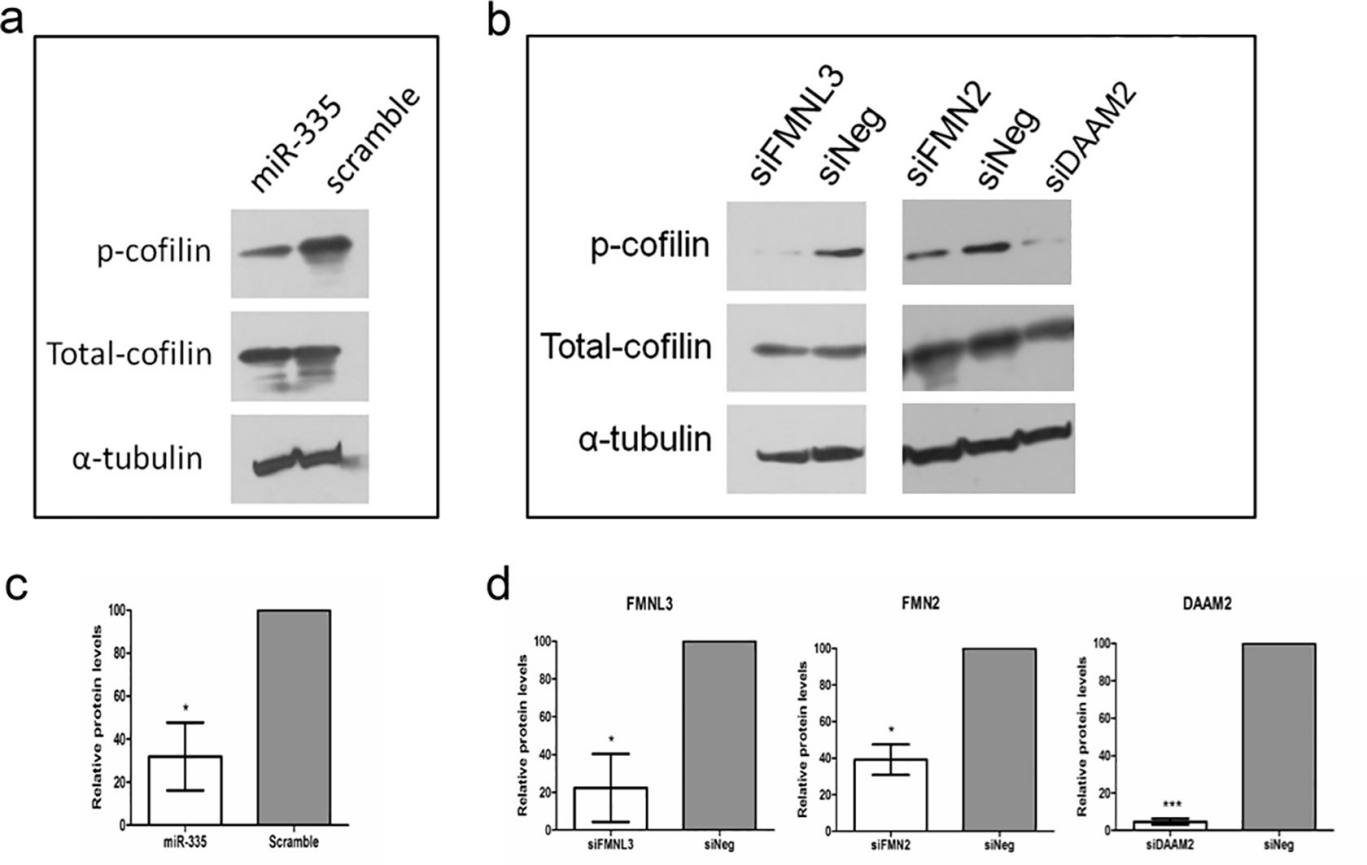
MiR-335, FMNL3, FMN2 and DAAM2 regulate actin cytoskeleton dynamics. (a) Transfection of cells with miR-335 mimics resulted in a reduction in the levels of phosphorylated cofilin protein and did not affect the levels of total cofilin protein (b) siRNA-mediated inhibition of *FMNL3*, *FMN2* and *DAAM2* produced a reduction in phosphorylated cofilin levels without altering total cofilin protein levels. Densitometric quantification of reduced phospho-cofilin protein in response to enhanced miR-335 expression (c) and reduced expression of *FMNL3*, *FMN2* and *DAAM2* (d) performed on duplicate blots and normalised against the appropriate alpha-tubulin loading control.

Specifically, the incorrect underlying data were used in Fig 2C for the FMN2 results and for the α-tubulin controls for the FMN1, FMN3, and FHOD3 experiments.

In Fig 5B, the siFMN2 experiment’s alpha-tubulin (flipped horizontally) was erroneously reported as the control data for the siDAAM2 experiment. The correct underlying data for these panels were published in S1 Fig and S5 Fig available with [1]. Note that the same siNeg control was used for the siFMN2 and siDAAM2 experiments, so this lane was intentionally duplicated in the original figure.

The corrected Fig 2 and Fig 5 are provided with this notice. The image reporting errors did not affect the densitometry analysis presented in Figs 2D, 5C and 5D. The individual level data underlying the Fig 2 and Fig 5 densitometry results are available in the S1 and S2 Files provided with this notice.

The authors apologize for any inconvenience and confusion these errors may have caused.

## Supporting information

S1 File Individual level data underlying densitometry analysis results presented in Fig 2. (XLSX)

S2 File Individual level data underlying densitometry analysis results presented in Fig 5 (XLSX)
