# Supplementary figures and images for: Metastasis Suppressor microRNA-335 Targets the Formin Family of Actin Nucleators
Source: PLoS One. 2013 Nov 5;8(11):e78428. doi: 10.1371/journal.pone.0078428 (PMC3818330; doi:10.1371/journal.pone.0078428)

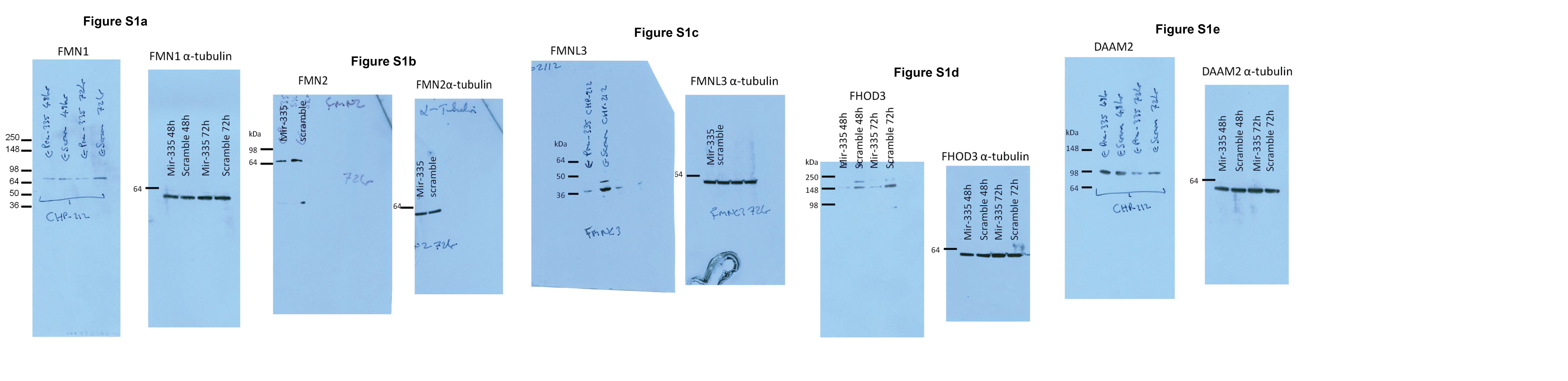

Supplement: Figure S1 — Full Western blots for data shown in Figure 2 . Molecular weight standards are displayed to the left of each blot. Each blot was subsequently striped and re-probed with alpha-tubulin (∼50 kDa) to serve as a loading control for normalisation and quantification of results. (S1a) CHP-212 cells transfected with either miR-335 or scramble control and probed with anti-FMN1 antibody which detects a band of approximately 85 kDa. (S1b) Full blot for FMN2 protein (∼67 kDa) and corresponding alpha-tubulin. (S1c) MiR-335 and scramble control transfected protein displaying reduction in FMNL3 protein levels (∼37 kDa). (S1d) Full blot image of FHOD3 protein (∼150 kDa) and corresponding alpha-tubulin control. (S1e) Western blot of DAAM2 protein (∼100 kDa) and equivalent loading control. (TIF) [file pone.0078428.s001.tif]

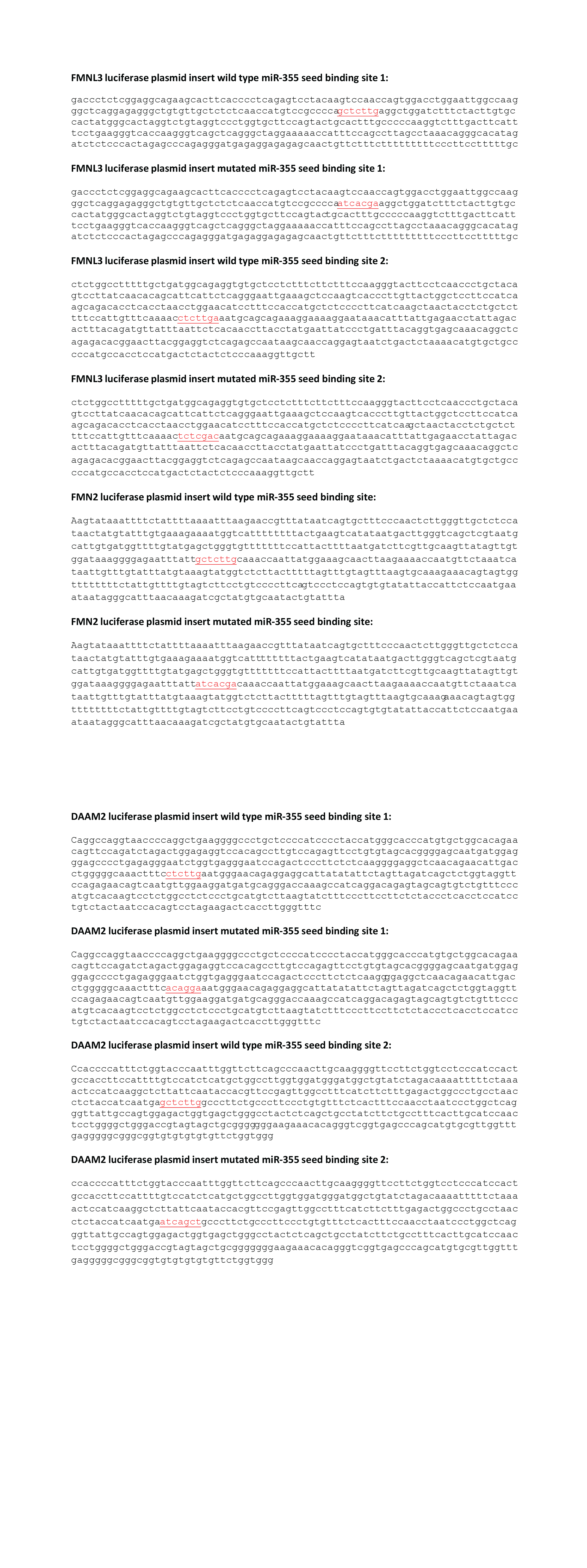

Supplement: Figure S2 — 3’UTR sequence inserts for FMNL3, FMN2 and DAAM2 . The miR-335 seed sequence match or mutated seed match are highlighted in red. (TIF) [file pone.0078428.s002.tif]

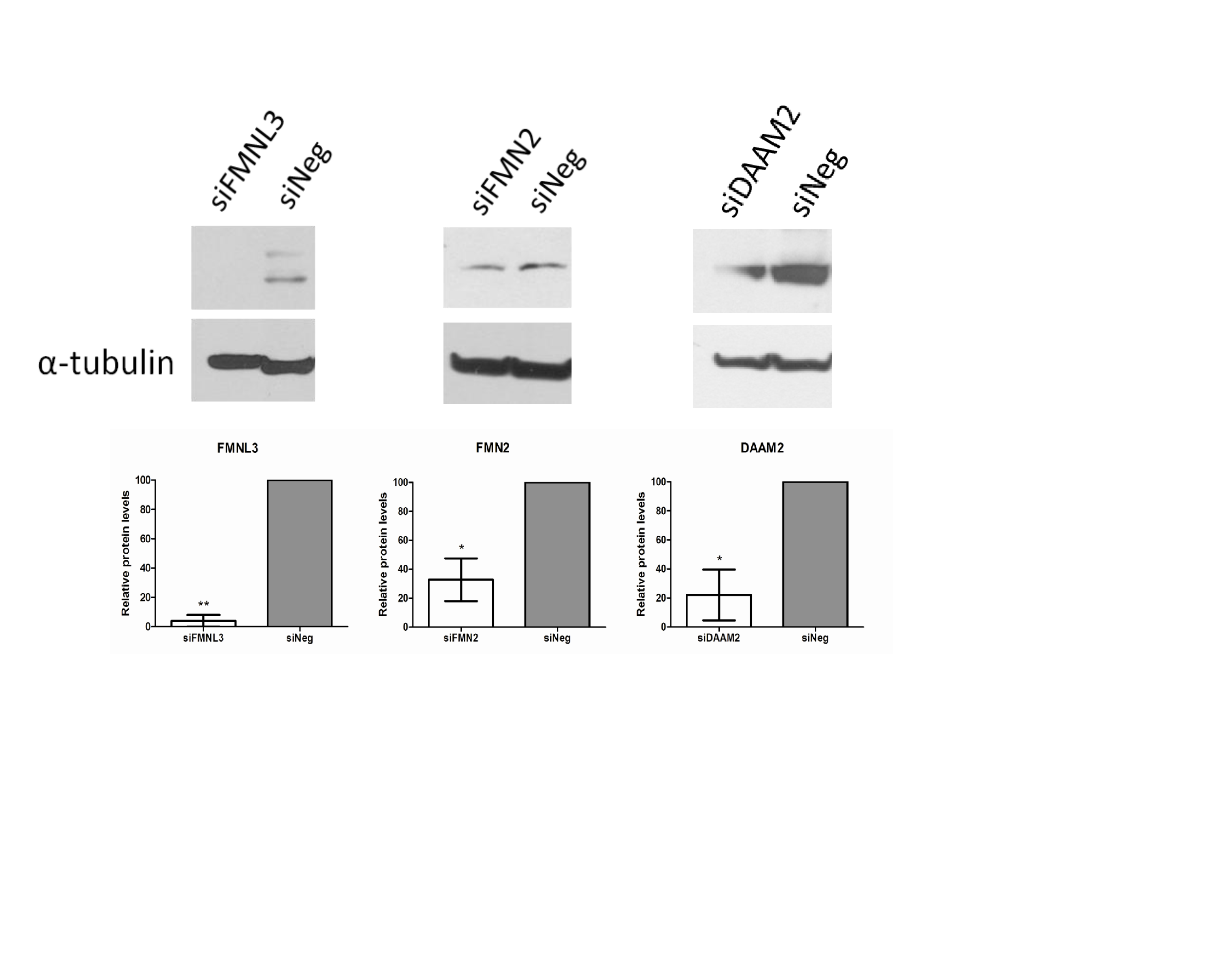

Supplement: Figure S3 — Western blot verification of siRNA knockdown of FMNL3, FMN2 and DAAM2. siRNA-mediated knockdown of FMNL3, FMN2 and DAAM2 produced significant reductions in the protein levels of each of the three formin genes by 72 hours post-transfection. Western blots were quantified by densitometric analysis of duplicate experiments. (TIF) [file pone.0078428.s003.tif]

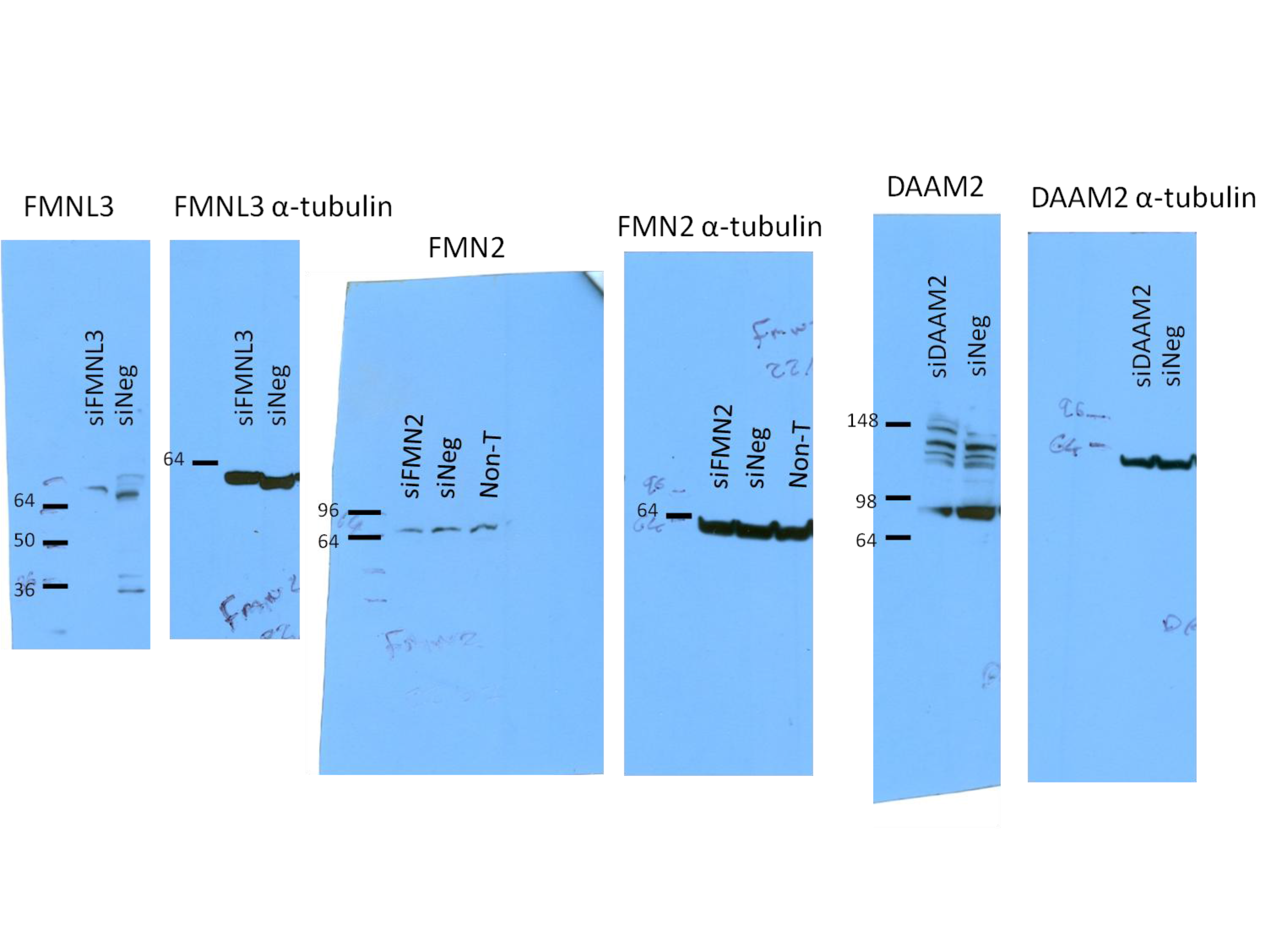

Supplement: Figure S4 — Full Western blot images corresponding to data displayed in Figure S3. (TIF) [file pone.0078428.s004.tif]

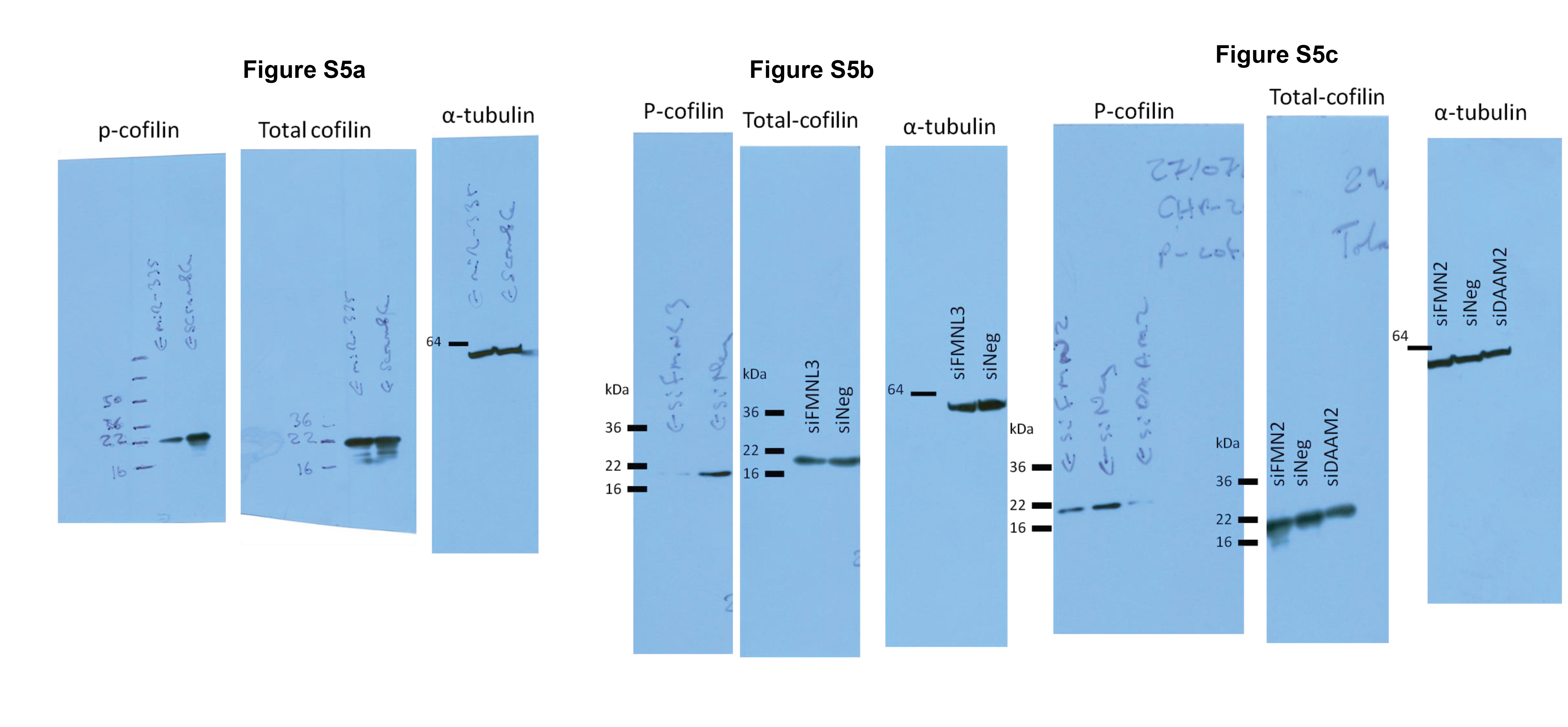

Supplement: Figure S5 — Full Western blot images for data displayed in Figure 5 . (S5a) Full blots of cells transfected with miR-335 or scramble control and analysed for levels of phosphorylated cofilin (∼19 kDa), subsequently re-probed for total cofilin protein levels (∼19 kDa) and finally re-probed for alpha-tubulin levels (∼50 kDa). (S5b) Cells transfected with siRNA to FMNL3 or siNegative control and analysed for phosphorylated cofilin, total cofilin and alpha-tubulin protein levels. (S5c) Cells transfected with siRNA to FMN2, DAAM2 or siNegative control and analysed for phosphorylated cofilin, total coffin and alpha-tubulin protein levels. (TIF) [file pone.0078428.s005.tif]

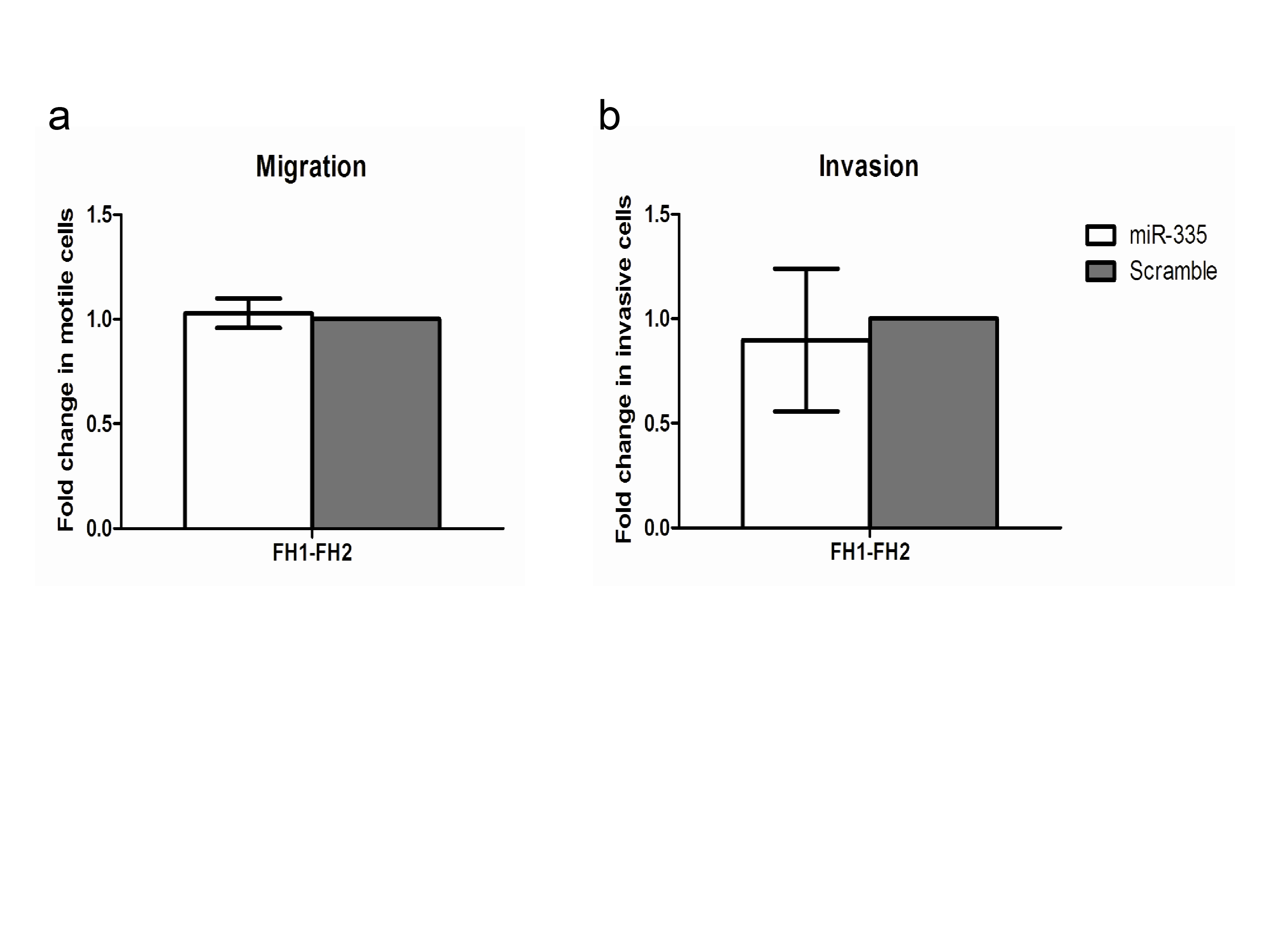

Supplement: Figure S6 — Analysis of co-transfection of FH1-FH2 FMNL3 in conjunction with miR-335 on cell migration and invasion. Co-transfection of SK-N-AS cells with the FH1-FH2 clone in addition to either miR-335 or scramble control had no significant effect on cell migration (a) or cell invasion (b). (TIF) [file pone.0078428.s006.tif]

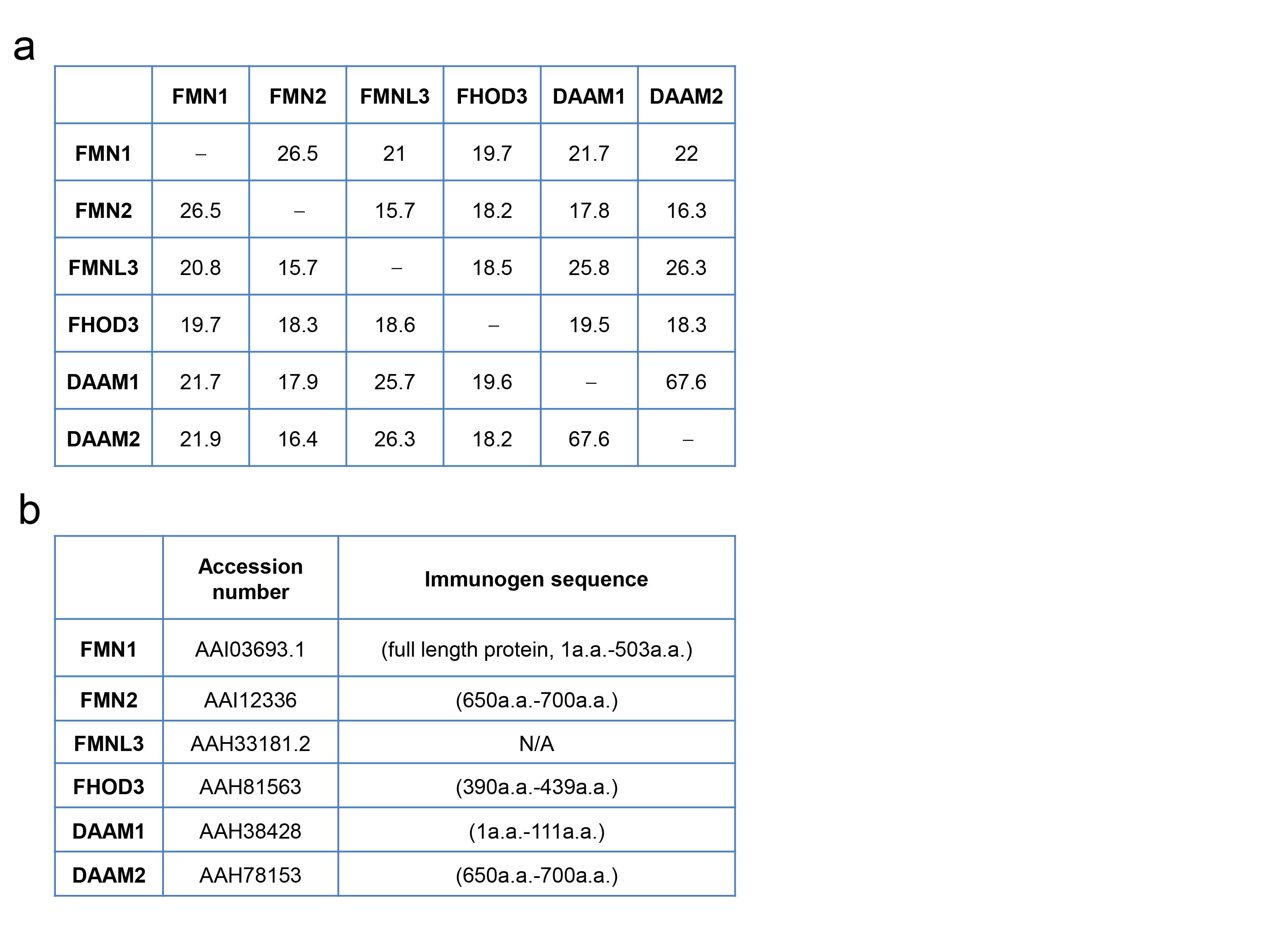

Supplement: Table S1 — Analysis of the specificity of the formin antibodies. (a) The accession number and immunogen sequence for each of the antibodies is displayed, the immunogen sequence for FMNL3 antibody was unattainable. The immunogen sequence of each of the formin antibodies was aligned to each of the five other formin member proteins. In the case of FMNL3, the alignment of the entire FMNL3 protein sequence with all five other protein members was analysed. None of the sequence alignments produced either continuous stretches of 9–13 or discontinues stretches of 15–22 amino acid complementarity, the defining criteria for peptide immunogenicity [41]. The only alignments that produced the required continuous amino acid complementarity for successful immunogenicity were DAAM1-DAAM2 sequence alignments. (b) The protein sequences of all six formin family members were aligned to each other and the percentage sequence similarity between each individual pair was calculated. (TIF) [file pone.0078428.s007.tif]
